# Chloroquine plays a cell-dependent role in the response to treatment of pancreatic adenocarcinoma

**DOI:** 10.18632/oncotarget.25745

**Published:** 2018-07-20

**Authors:** María Inés Molejon, Mirna Swayden, Daniele Fanale, Jennifer Bintz, Odile Gayet, Philippe Soubeyran, Juan Iovanna

**Affiliations:** ^1^ Centre de Recherche en Cancérologie de Marseille (CRCM), INSERM U1068, CNRS UMR 7258, Aix-Marseille Université and Institut Paoli-Calmettes, Parc Scientifique et Technologique de Luminy, Marseille, France; ^2^ INCITAP-CONICET (Instituto de Ciencias de la Tierra y Ambientales - Consejo Nacional de Investigaciones Científicas y Técnicas), Facultad de Ciencias Exactas y Naturales, Universidad Nacional de La Pampa (UNLPam), Santa Rosa, La Pampa, Argentina; ^3^ Department of Surgical, Oncological and Oral Sciences, Section of Medical Oncology, University of Palermo, Palermo, Italy

**Keywords:** pancreas cancer, autophagy, Chloroquine, gemcitabine

## Abstract

In this study, our aim is to assess the role played by autophagy and its inhibition in the different PDAC cellular compartments, and its involvement in chemo-resistance using primary human pancreatic cancer-derived cells (PCC) and Cancer Associated Fibroblasts (CAF). Autophagy flux, as measured by LC3-I and -II in the presence of Chloroquine, showed a variable level in PCC and CAFs. We found no correlation between autophagy level and degree of tumor differentiation. Association of Chloroquine with gemcitabine, 5FU, oxaliplatin, irinotecan and docetaxel revealed that its effect on survival is cell- and drug-dependent *in vitro* and *in vivo*. In addition, we demonstrated that autophagy in CAFs can play an important role in sensitizing PDAC to anticancer treatments since its inhibition increased the resistance of PCCs to gemcitabine. In conclusion, this work clearly shows a heterogeneity in the effect of Chloroquine and highlights a role of CAFs autophagy in sensitizing tumors to treatments. It also reveals that the role of autophagy is more complex than expected in PDAC as well as its sensitivity to treatments.

## INTRODUCTION

Pancreatic ductal adenocarcinoma (PDAC) is a highly lethal disease due to early metastasis, rapid evolution, and strong chemoresistance [1]. PDAC cells show an extreme resistance to current therapeutic treatments, probably due to altered mechanisms of cell survival and metabolic pathways [2]. In PDACs, the abundant stroma produced by Cancer-Associated Fibroblasts (CAFs), act as a mechanical barrier against the effective delivery of chemotherapeutic agents [3]. In addition to the secreted ECM components, CAFs have been described to be important for cell survival and metastatic signaling, thereby promoting tumor growth and invasion. This is mainly carried out through the activation of basic cellular processes, such as autophagy [4]. However, the failure of Sonic Hedgehog (SHH) inhibitor vismodegib [5] and LOX inhibitor [6] in clinical trials suggests the need to revise this paradigm.

Autophagy is a catabolic process of degrading organelles and macromolecules, to allow the recycling of energetic products [7]. Autophagy modulates cellular metabolism [8], and its increased rate has been associated with cancer development and aggressiveness [9]. This process is activated in response to stress, such as nutrient deprivation, in order to promote cell survival. Emerging evidences have shown that autophagy may play an important role in the regulation of chemoresistance to anti-tumoral treatments [10, 11]. However, the role of autophagy in the regulation of tumor development and drug resistance is still elusive. Many studies suggest that autophagy plays a protective role, favoring tumor development, rather than a suppressive function [12, 13]. However, it has been described that this process may have a tumor promoting or an inhibitory function depending on the tumor compartment in which it is activated [14]. So, this is why it is important to understand the role of autophagy in each tumor compartment separately. Drugs effectively inhibiting autophagy flux are available, one of the most efficient being the anti-malarial drug Chloroquine [15]. This drug is approved by the FDA and it inhibits autophagy at its late stages by preventing the lysosomal acidification [16].

In this study, we show that the effect of the treatment of PDAC cells with Chloroquine, in combination with five anticancer drugs, is tumor- and drug-dependent. Moreover, we show that blocking autophagy in CAFs increases the resistance PDAC-derived cells to gemcitabine based chemotherapy.

## RESULTS

### Autophagy levels heterogeneity in PDAC primary cell cultures and CAFs

**Figure 1:**
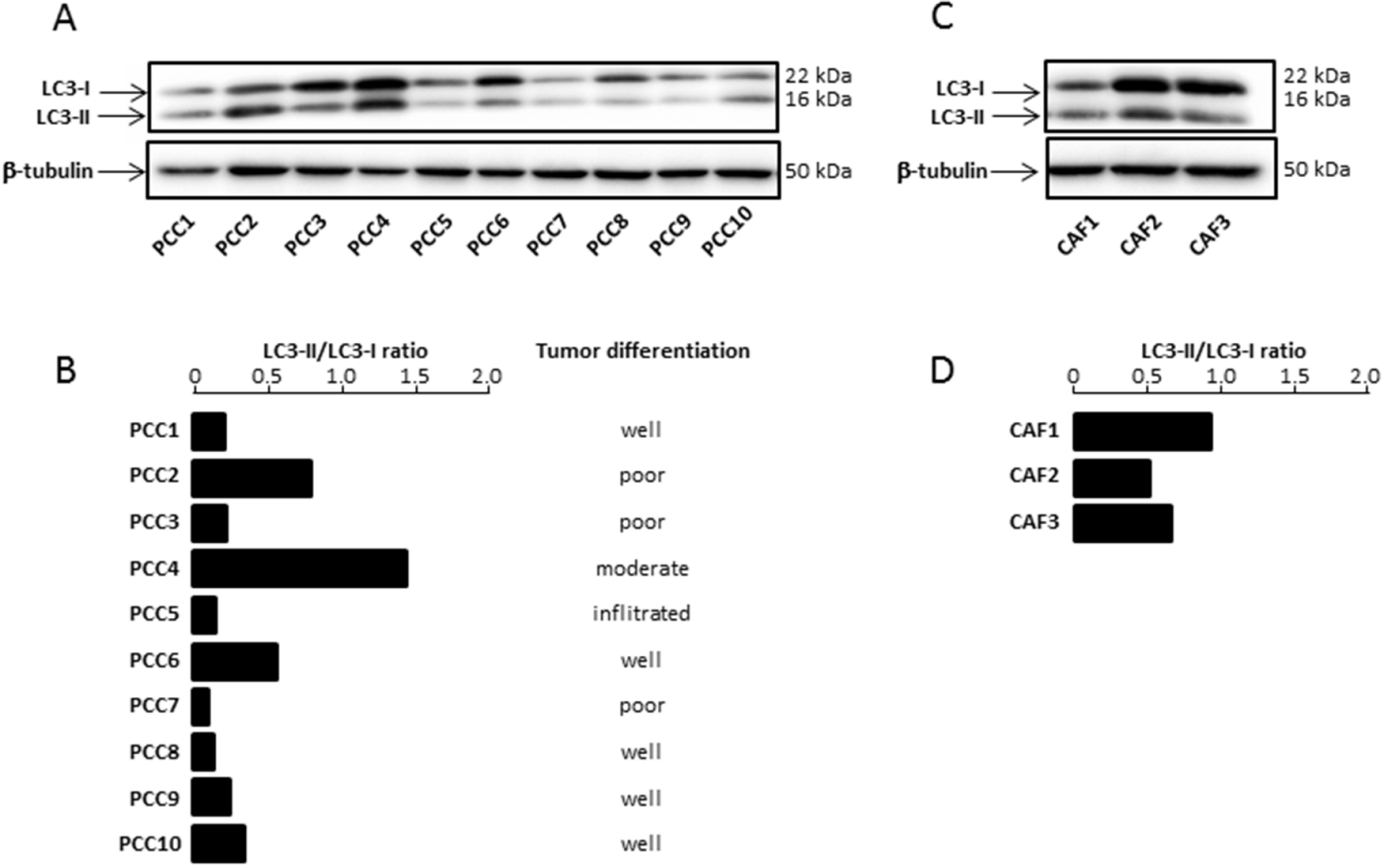
**(A)** Basal Autophagy was evaluated by proteolysis of LC3-II by western blot. **(B)** The bar graph indicates the ratio between LC3-II and LC3-I at basal state after β-tubulin normalization. Autophagy levels were estimated and compared with tumor grade differentiation. **(C)** Basal Autophagy was evaluated in three CAFs. **(D)** The bar graph indicates the ratio between LC3-II and LC3-I at basal state after β-tubulin normalization. Experiments were repeated at least 2 times.

The role of autophagy in cancer is complex and varies among the tumors types and the stage of the disease [17]. With the aim to explore the role of this biological process in PDAC chemoresistance, we first evaluated autophagy in 10 PDAC primary cells (PCC) derived from PDTX (Patients Derived Tumor Xenografts). The microtubule-associated protein 1 light chain 3-II (LC3-II), a mammalian orthologous of Atg8, is a specific autophagy marker largely utilized for measuring the autophagy level in cells and tissues [18]. We have measured the LC3II/LC3I ratio of PCCs grown under nutrient-rich conditions to evaluate their basal level of autophagy and we could observe that it varies widely between the cell lines (Figure 1A), thereby confirming the heterogeneity of autophagy rates between different pancreatic patient tumors. We then analyzed the association between autophagy levels of the PCC along with their differentiation score. We found no significant correlation between degree of differentiation of the PCCs and their autophagy levels (Figure 1B). Next, we have evaluated the autophagy occurring in three human pancreatic cancer-derived CAFs (CAF1, CAF2 and CAF3). Similarly, our results showed a variation of basal autophagy levels among the three different CAFs tested (Figure 1C, 1D).

**Figure 2:**
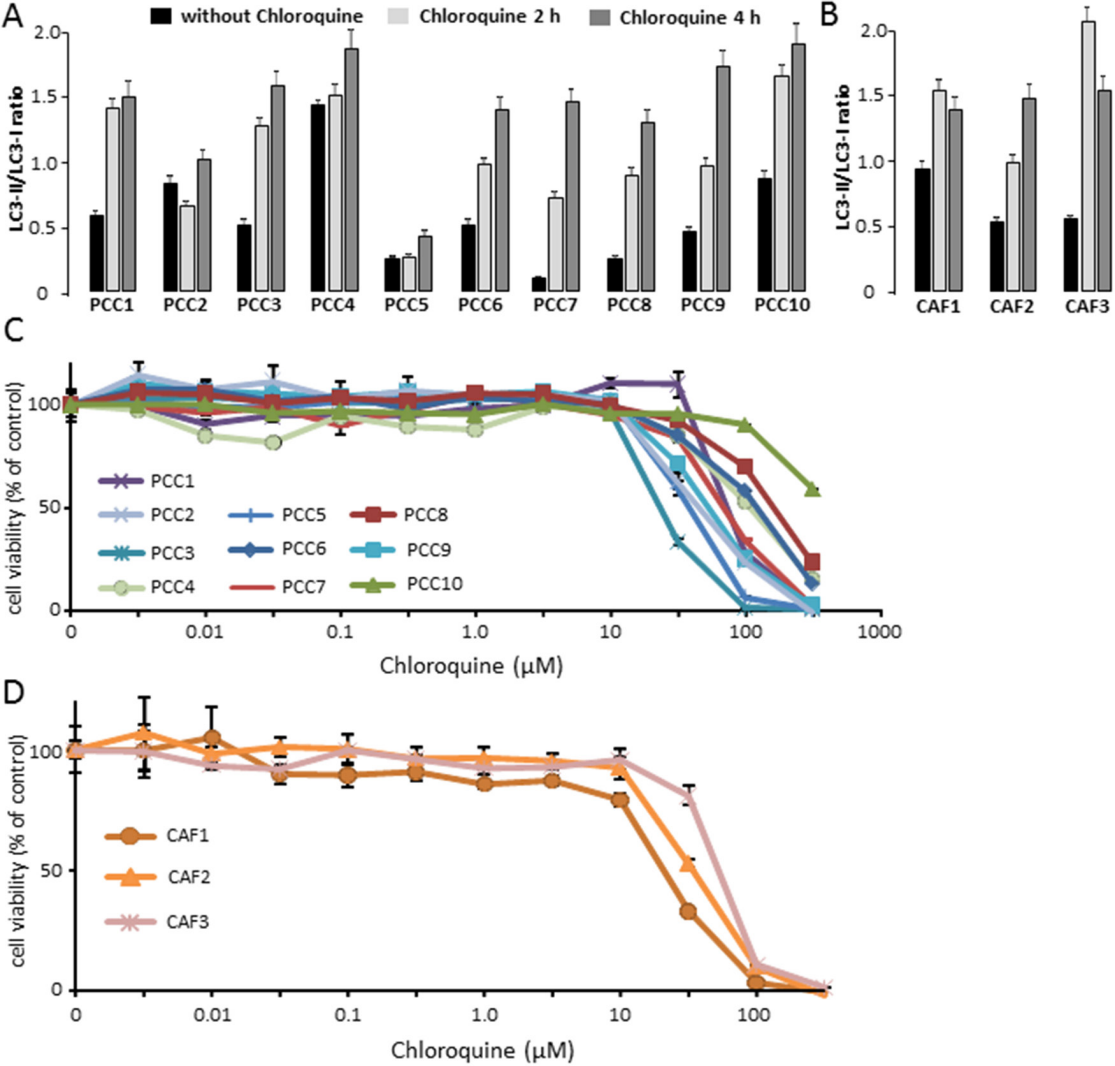
**(A)** PCCs treated with 10 μM Chloroquine for 2 h or 4 h, and were compared with control. The estimated ratio of LC3-II/LC3-I was calculated between 2 h and 4 h Chloroquine treatment and basal state. **(B)** Three different CAFs were treated with 10 μM Chloroquine for 2 h and 4 h, and the ratio of LC3-II/LC3-I was compared with control. **(C)** PDAC-derived primary cells were treated with increasing concentrations of Chloroquine and the cells viability were measured after 72 h of treatment. **(D)** CAFs were treated with increasing concentrations of Chloroquine and the cells viability were measured after 72 h of treatment. Error bars ± SEM; n = 3 per group. Experiments were repeated at least 2 times.

It has been previously described that autophagy is required for the development of PDAC established cell lines in xenografts [19]. Chloroquine is a lysosomotropic agent that inhibits auto-lysosomal clearance [20] and it has been previously described that in combination with other chemotherapeutic agents, Chloroquine enhances their anti-cancer effects in several tumor types [21, 22]. Hence, we have evaluated the effect of Chloroquine treatment in both primary PDAC derived cells and CAFs. First, we confirmed that Chloroquine could block the autophagic flux in our studied cells. This was carried out by treating 10 primaries PCCs and 3 CAFs with 10 μM Chloroquine for 2 h and 4 h followed by measurement of LC3-II and LC3-I levels by western blot. Our results showed that Chloroquine treatment increased the ratio of LC3-II/LC3-I after 2 h and 4 h of treatment in all analyzed cells (Figure 2A and 2B). Then, we tested the relative sensitivity of PCCs and CAFs to increasing concentrations of Chloroquine by measuring the percentage of viable cells at each concentration. Our results showed that the IC_50_ of primary PCCs tested showed a wide variability (from 50 to 500 μM), which confirmed a strong heterogeneity in the response to Chloroquine between different PDAC samples (Figure 2C). Similar results were obtained with the three CAFs tested (Figure 2D) which displayed an IC_50_ of 40 μM, 60 μM and 80 μM.

### Chloroquine treatment plays a cell-dependent role when combined with chemotherapeutic drugs *in vitro*

**Figure 3:**
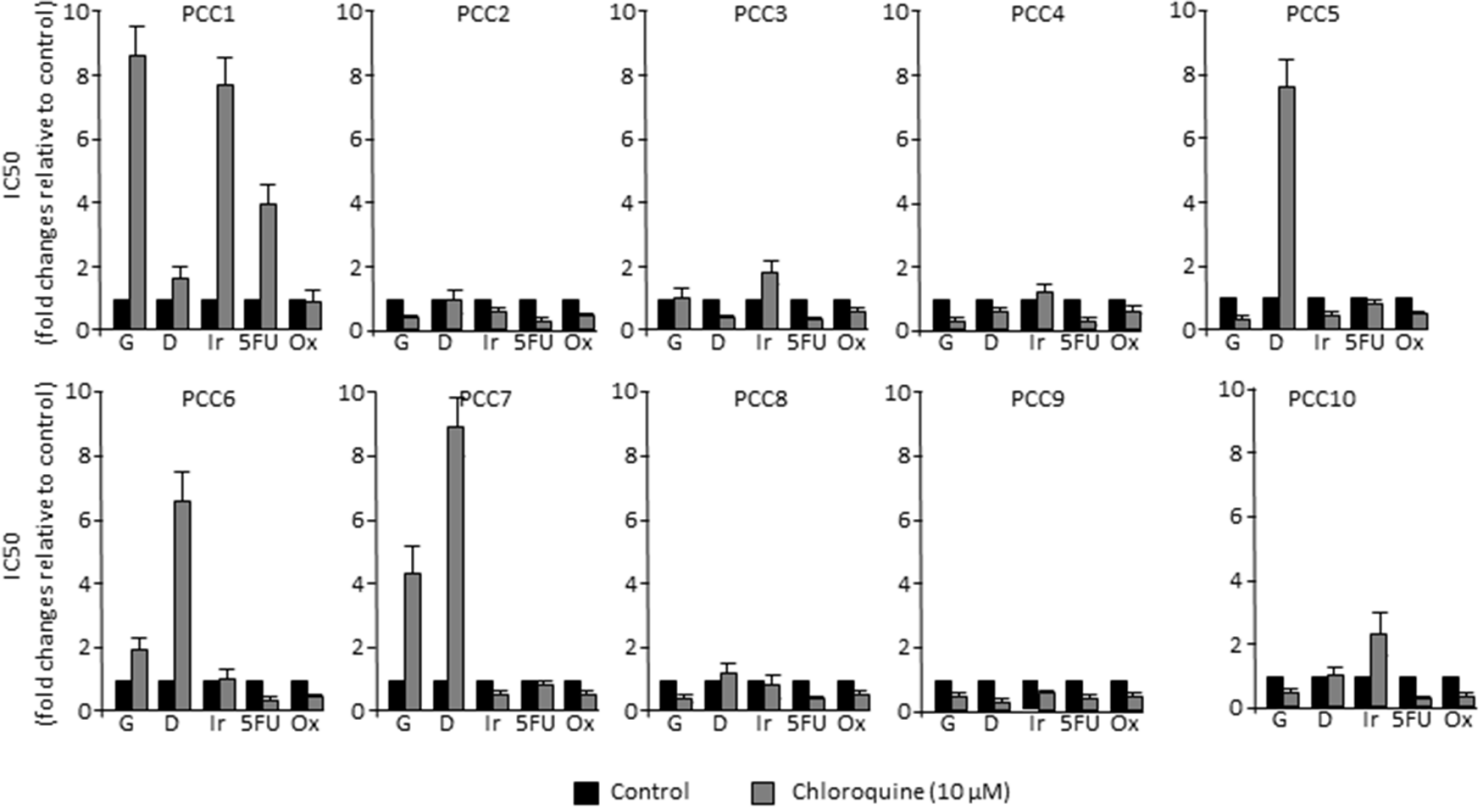
PDAC-derived cells response to chemotherapy *in vitro* Each primary cell line was treated with increasing concentrations (from 0 to 1000 μM) of gemcitabine (G), docetaxel (D), 5-Fluouracil (5FU), Oxaliplatin (Ox) and Irinotecan (Ir) alone or in combination with 10 μM Choloroquine. Cell survival was measured after 72 h of treatment. The profile of sensitivity was obtained for each drug and the IC_50_ in response to each drug, with or without Chloroquine, is presented in the bar graphs relative to control.

Autophagy is a mechanism that is essential for cell survival in response to several types of stresses. In fact, when cancer cells are subjected to stress conditions, autophagy is activated in order to maintain the cellular homeostasis [23, 24]. On the contrary, it has been suggested that the impairment of autophagy can lead to tumorigenesis in PDAC [19]. Hence, we aimed to evaluate the tumor response to the autophagy flux inhibition by Chloroquine in combination with the five most common chemotherapeutic drugs used in the treatment of PDAC. This was carried out by treating 10 primary PCCs derived from PDTXs with increasing concentrations of gemcitabine (G), 5-Fluouracil (5FU), oxaliplatin (Ox), Irinotecan (Ir) and docetaxel (D), alone or in combination with 10 μM of Chloroquine. Then, we measured cell viability after 72 h of treatment. Surprisingly, our results demonstrated that the effect of combining Chloroquine with chemotherapy was drug and cell type dependent. Interestingly, whereas Chloroquine increased sensitivity of some cells to the treatment, other tended to be more resistant while others were found to be insensitive (Figure 3). Accordingly, no correlation was found between autophagy flux levels of PCCs and their sensitivity to each of the five chemotherapeutic drugs used (Table 1), indicating that a higher level of autophagy does not necessarily account for a better response to the chemotherapeutic drugs.

**Table 1:** Linear regression was calculated between the IC_50_ for each treatment and levels of basal autophagy

	G	G+CQ	D	D+CQ	Ir	Ir+CQ	5FU	5FU+CQ	Ox	Ox+CQ
R^2^ LC3II/LC3I	0.04	0.008	0.12	0.12	0.0008	0.01	0.04	0.04	0.44	0.11

gemcitabine (G), docetaxel (D), 5-Fluouracil (5FU), Oxaliplatin (Ox) and Irrinotecan (Ir) alone or in combination with Choloroquine (CQ).

### Chloroquine influences the effect of gemcitabine on tumor growth in a cell-dependent manner *in vivo*

**Figure 4:**
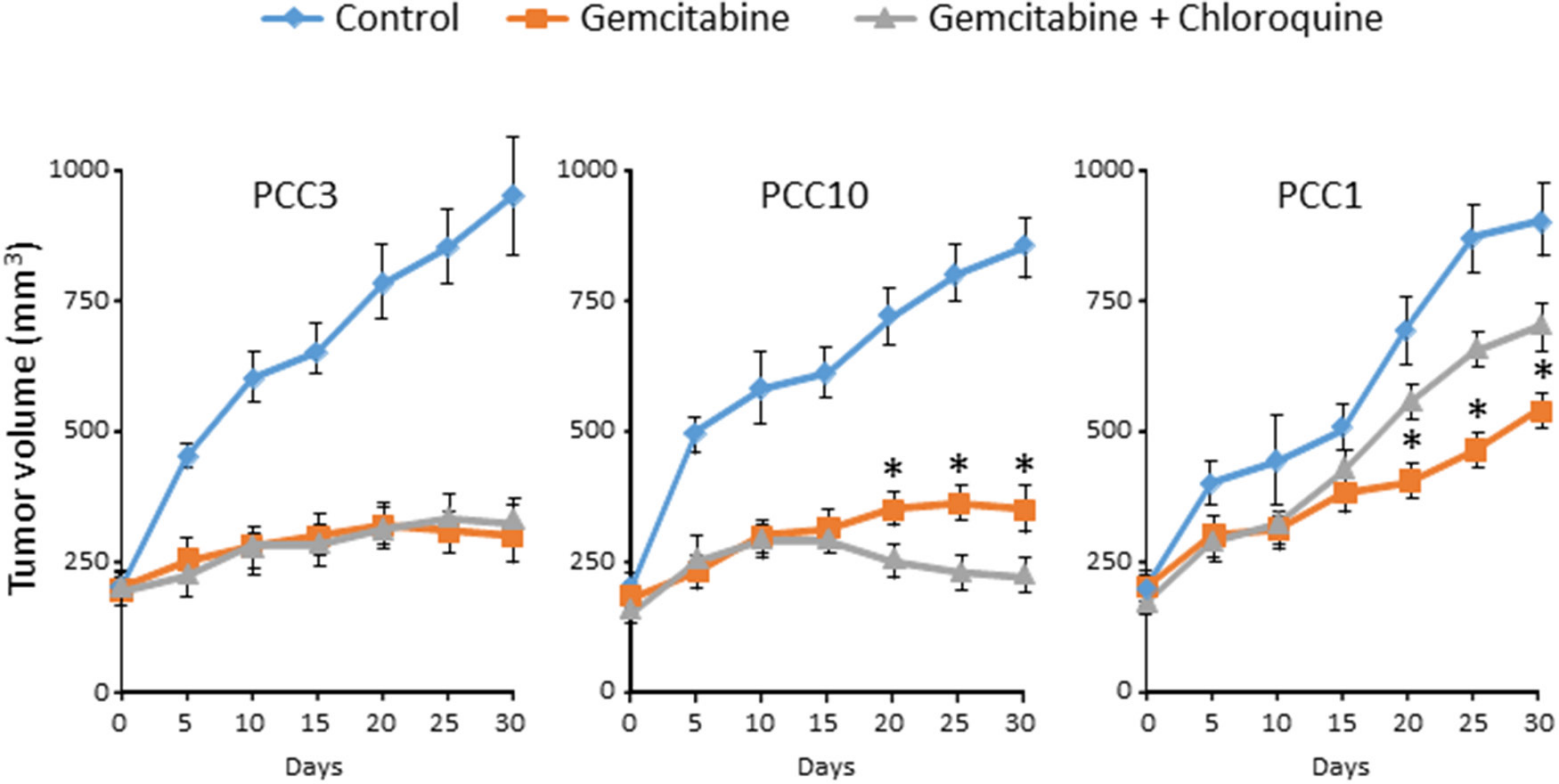
PCCs xenografts were treated with vehicle or gemcitabine alone or in combination with Chloroquine (50 mg/kg/day) after tumors attain a volume of 200 mm^3^ The tumor volume was monitored every 5 days. Error bars ± SEM; n=5 per group. ^*^P<0.05 compared to Chloroquine treated.

Our *in vitro* results suggested that Chloroquine influences the effect of anticancer drugs in a cell- and drug-dependent manner. Therefore, we evaluated the effect of Chloroquine *in vivo* using three different PCCs selected according to their response to the combination of gemcitabine with Chloroquine, positive, negative or null (see Figure 3). Interestingly, the effect of Chloroquine was consistent with the *in vitro* observations. It was insignificant in PCC3 xenograft, whereas it increased the sensitivity to Gemcitabine in PCC10 and increased the gemcitabine resistance in PCC1 (Figure 4). Altogether, our *in vitro* and *in vivo* results strongly suggest that Chloroquine may have no effect, or could increase or decrease the sensitivity of PCCs in a cell- and drug-dependent manner.

### Chloroquine treatment influences the chemoresistance of CAFs

**Figure 5:**
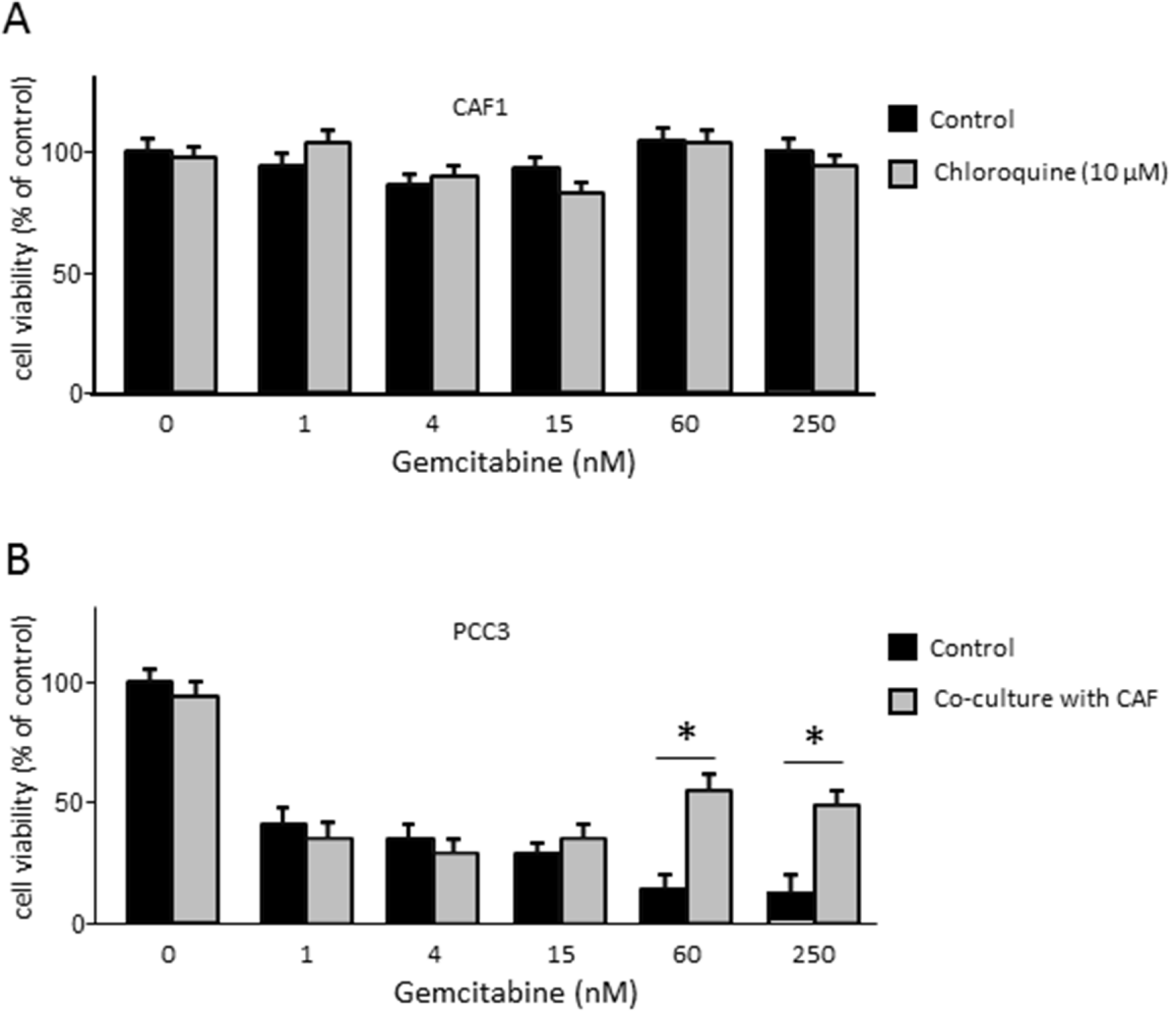
**(A)** Evaluation of the CAFs viability under increasing doses of gemcitabine in cells previously treated or non-treated treated with 10 μM of Chloroquine. **(B)** Proliferation of PCC3 co-cultured with CAFs previously treated or not treated with 10 μM Chloroquine for 48 h. PCCs were added to the upper part of the transwells and were incubated with increasing concentrations of gemcitabine for 24 h. Error bars ± SEM; n = 3 per group. ^*^P<0.05 compared to control. Experiments were repeated at least 2 times.

As mentioned above, autophagy may have a tumor promoting or an inhibitory role depending on the cell compartment in which it is activated [14]. Therefore, it is important to understand the role of autophagy in the resistance to the treatments in each tumor compartment separately. We have shown that autophagy occurring in cancer cells did not influence the efficacy of chemotherapy. Therefore, we aimed to assess the involvement of CAFs’ autophagy in the chemoresistance of PDAC-derived cells. First, we subjected CAFs treated or not with 10 μM of Chloroquine to increasing doses of gemcitabine and found that these treatments did not affect the viability of CAFs (Figure 5A). Then, we performed a co-culture assay that allowed us to separate autophagy induction in CAFs from the one in PCCs. We co-cultivated CAFs previously treated with 10 μM of Chloroquine for 48 h with PCCs, and subjected them to increasing doses of gemcitabine for 24 h. Our results showed that under lower doses of gemcitabine (below 15 nM) the sensitivity of PCCs was not affected upon cultivation with Chloroquine-treated CAFs (Figure 5B). However, under higher doses of gemcitabine (60 nM or more), the percentage of cell viability was higher when PCCs were cultivated with Chloroquine-treated CAFs (Figure 5B). Hence, blocking autophagy in CAFs increased the resistance of PCCs to gemcitabine treatment, suggesting that autophagy in CAFs can contribute in sensitizing PDAC to chemotherapy.

## DISCUSSION

Autophagy is shown to be a mechanism that promotes cell survival or can act in parallel with cell death when it is inhibited [25]. Therefore, autophagy inhibitors combined with chemotherapies are widely used as a cancer treatment [26]. However, the study of the ability of autophagy inhibitors to overcome resistance to anticancer therapies raises many questions. In the present work, we show for the first time that autophagy levels in human PDAC-derived cells are not correlated with tumor differentiation scores, suggesting that higher levels of autophagy are not correlated with a more differentiated and a less aggressive tumor phenotype. Furthermore, we found that blocking autophagy with Chloroquine, a commonly used inhibitor, may improve cancer therapies, but in a cell- and drug-dependent manner as presented in Figures 3 and 4. Despite supportive preclinical data for the combination of Chloroquine with gemcitabine [27], results here showed that in some cases there is no improvement of treatment efficacy by adding autophagy inhibitors compared to the single-agent therapy. This outcome raises concerns about the role of autophagy in cancer development. It has been proposed that autophagy is activated as a protective mechanism during chemotherapy, but it has also been implicated in the induction of autophagic cell death.

Autophagy dependency and metabolic stress levels of tumour cells vary widely depending on the tumor type and progression stage [28]. Therefore, reliable measurements to predict tumour sensitivity to autophagy inhibition would be extremely useful for patient selection in clinical practice [29]. As it was previously described, the status of tumour suppressor p53 can affect Chloroquine efficacy, and this may explain the dual effect found after Chloroquine therapy. Moreover, other studies have observed that higher steady-state mitochondrial membrane potential values, representing mitochondrial stability, can predict cancer cell resistance to Chloroquine treatment [30]. Thus, it appears that, depending on cancer types and cell state, autophagy differently impacts the effect of anticancer drugs on cell survival. Numerous clinical trials in which Chloroquine is being used to treat patients with a range of cancer types are registered in clinical trial databases, although some few trials have been completed, so limited published data are available. Nevertheless, there are many *in vivo* research which supported our findings [31–36]. Possibly, in some PDAC cells the role played by autophagy can be anti-apoptotic but pro-apoptotic in other cell types, therefore the consequences of inhibiting the autophagic flux is dependent on the biology of the PDAC cells.

Stroma in PDAC is composed mainly of CAFs and inflammatory cells. Previous studies have suggested that the stroma acts as a barrier influencing the delivery of chemotherapeutic compounds to the tumor [37]. Nevertheless, preclinical studies have demonstrated that depletion of cancer-associated fibroblasts failed to improve the efficacy of therapies and, in contrary, resulted in an accelerated progression of the disease [6]. Here, we found that blocking autophagy in CAFs did not affect their viability upon gemcitabine treatment but, surprisingly, could increase the resistance of co-cultivated PDAC cells when subjected to high doses of the same drug (Figure 5B). Because in our experimental model both cancer cells and CAFs are not in direct contact therefore we assume that the only possibility to affect sensitivity to treatments is through released factors. Accordingly, several mechanisms, including release of the exosomes and secretion of soluble factors, have been attributed to CAFs for increasing the resistance to treatments. In this way, after autophagy inhibition we can suppose that both release of exosomes and secretion of soluble factors increased. Hence, it appears the CAFs autophagy is necessary to preserve the efficacy of gemcitabine over cancer cells and this is probably one mechanism responsible for the bi-therapy failure.

In conclusion, we have demonstrated that inhibition of autophagic flux in PDAC-derived cells, using Chloroquine, influences the effect of the anticancer drugs in a cell- and drug-dependent manner. Therefore, it will be necessary to identify the appropriate markers that will determine when the Chloroquine treatment will increase the sensitivity of PDAC to a specific anticancer drug. Finally, this work also demonstrates that autophagy in CAFs plays an important role in sensitizing PDAC to anticancer treatments. Finally, although this work clearly shows the very high heterogeneity in the effect of Chloroquine and highlights the importance of CAFs autophagy in sensitizing tumors to treatments, it also reveals that the role of autophagy in PDAC is more complex than expected regarding the development of the disease and regarding the response to treatments.

## MATERIALS AND METHODS

### Tumor samples

Patients were recruited for this study under the Paoli Calmettes Institute clinical trial number 2011-A01439-32. After patients were informed, consent had been obtained, excess tissue samples from resected PDACs were collected for xenograft or cell lines procedures. The tumor tissue used for xenograft development was deemed excess to that required for the patient's diagnosis and standard of care and treatment. Two types of samples were obtained, Endoscopic Ultrasound-Guided Fine-Needle Aspiration (EUS-FNA) biopsies from patients with unresectable tumors, and tumoral tissues from patients undergoing surgery. Each sample obtained from EUS-FNA was mixed with 100 μl of Matrigel (BD Biosciences) and injected in the upper right flank of a nude mouse (Swiss Nude Mouse Crl: NU(lco)-Foxn1nu, Charles River Laboratories). Each sample derived from surgery resection was fragmented, mixed with 100 μl of Matrigel (BD Biosciences) and implanted with a trocar (10 Gauge, Innovative Research of America, Sarasota, FL) in the subcutaneous right upper flank of an anesthetized and disinfected mouse. When tumors reached 1 cm^3^, mice were sacrificed and removed. Xenografts that failed to develop within 6 months were stopped. Patient anonymity was maintained by removing any information that could lead to the identification of the patient. Post-surgical anatomopathology reports were provided for specimens from each patient. Histopathologic evaluation was performed on 5-μm H&E-stained sections of patient tumors and pathologists determined tumor differentiation score.

### Animal experiments

All animal experiments were conducted in accordance with institutional guidelines and were approved by the “Plateforme de Stabulation et d'Expérimentation Animale” (PSEA, Scientific Park of Luminy, Marseille). Briefly, a total number of 10 human PDAC xenografts were established. Tumor specimens (100 mm^3^), from resected PDAC patients, were mixed with Matrigel (BD Biosciences) and implanted subcutaneously on the upper right flank of 5- to 6-week-old nude mice (Swiss Nude Mouse Crl: NU (lco)-Foxn1nu, Charles River Laboratories). Tumor size and body weights of all animals were measured weekly. All mice were divided into groups receiving injections of PCC alone (1 × 10^6^ per mouse); PCCs treated with gemcitabine and PCCs receiving gemcitabine in combination with Chloroquine. Subcutaneous tumor measurements were undertaken using calipers and values were calculated as (length x width^2^)/2. Gemcitabine (Lilly) treatment was administered twice weekly (100 mg/kg; i.p.). Chloroquine was administered daily (50 mg/kg; i.p.)

### Immunoblotting

Protein extraction was performed, on ice, using total protein extraction buffer: 50 mM HEPES (pH 7.5), 150 mM NaCl, 20% SDS, 1 mM EDTA, 1 mM EGTA, 10% glycerol, 1% Triton, 25 mM NaF, 10 mM ZnCl_2_ and 50 mM DTT. Before lysis, protease inhibitor cocktail at 1:200 (Sigma-Aldrich; NUPR1340), 500 mM PMSF, 1 mM sodium orthovanadate and 1 mM β-glycerophosphate were added. Protein concentrations were measured using a BCA Protein Assay Kit (Pierce Biotechnology). Protein samples (80 mg) were denatured at 95°C and subsequently separated by SDS-PAGE gel electrophoresis. After being transferred to nitrocellulose, the membrane was blocked with 1% BSA, and the samples were probed with primary antibody, followed by a horseradish peroxidase-coupled (HRP) secondary antibody. β-Tubuline antibody was used as a loading control.

### Cell culture

For *in vitro* studies, tumor fragments were enzymatically digested with collagenase type V (Sigma) and trypsin/EDTA (Gibco, Life Technologies) and suspended in DMEM, supplemented with 1% (w/w) Penicillin/Streptomycin (Gibco, Life Technologies) and 10% Fetal Bovine Serum (Lonza). After centrifugation, cells were re-suspended in Serum Free Ductal Media (SFD), adapted from Schreiber et al [38], at 37°C in a 5% CO_2_ incubator. Cells were deprived of antibiotics at least 48 h before performing tests.

Cancer-associated fibroblasts (CAFs) were prepared by the outgrowth method. Fresh tissue was obtained from residual pancreatic adenocarcinoma specimens from patients undergoing primary surgical resection. All human samples were obtained in accordance with the policies and practices of the Paoli Calmettes Institute clinical trial number 2011-A01439-32. Briefly, tumor samples were minced and seeded in six-well plates containing 15% FCS/DMEM, L- glutamine (2 mmol/L), penicillin/streptomycin, and amphotericin. After 25 days, cells were able to grow out from the tissue clumps. Medium was changed every 3 days. All cells were maintained at 37°C in a humidified atmosphere of 5% CO_2_.

### Chemograms

Cell chemosensitivity was assessed using gemcitabine 5-fluorouracil (5-FU), oxaliplatin, docetaxel and irinotecan, and Chloroquine (Sigma). Five thousand cells per well were plated in 96-wells plates in SFD media. 24 h later the media was supplemented with increasing concentrations of drugs (0 to 1000 μM), and incubated for an additional 72 h period. Each experiment was done in triplicate and repeated at least two times. Cell viability was measured after incubation with the PrestoBlue reagent (Life Technologies) for 3 h, the following the PrestoBlue cell viability reagent protocol provided by the supplier.

### Co-culture

CAFs were seeded at a density of 2.5 × 10^5^ cells per well in a 6-co-culture well. 1.5 × 10^5^ cells Pancreatic Cancer cells (PCC) were seeded in the insert well (0.4 micron). 24 h later, CAFs were either treated with 10 μM of Chloroquine or with vehicle. After 4 h incubation, the transwells containing PCCs were added to the plate, which were previously treated with increasing concentrations of gemcitabine. After 48 h of co-culture, cells were trypsinized and their viability analyzed.

### Statistical analysis

Results for continuous variables are expressed as means ± standard error of the mean (SEM). Overall comparisons of continuous variables were performed using the unpaired two-tailed Student's t-test. All tests of significance were two-tailed and the level of significance was set at 0.05. All data are representative of at least two independent experiments.

## References

[1] Paulson AS, Tran Cao HS, Tempero MA, Lowy AM (2013). Therapeutic advances in pancreatic cancer. Gastroenterology.

[2] Grasso C, Jansen G, Giovannetti E (2017). Drug resistance in pancreatic cancer: impact of altered energy metabolism. Crit Rev Oncol Hematol.

[3] Olive KP, Jacobetz MA, Davidson CJ, Gopinathan A, McIntyre D, Honess D, Madhu B, Goldgraben MA, Caldwell ME, Allard D, Frese KK, Denicola G, Feig C (2009). Inhibition of Hedgehog signaling enhances delivery of chemotherapy in a mouse model of pancreatic cancer. Science.

[4] Kim KH, Lee MS (2014). Autophagy—a key player in cellular and body metabolism. Nat Rev Endocrinol.

[5] Catenacci DV, Junttila MR, Karrison T, Bahary N, Horiba MN, Nattam SR, Marsh R, Wallace J, Kozloff M, Rajdev L, Cohen D, Wade J, Sleckman B (2015). Randomized Phase Ib/II Study of Gemcitabine Plus Placebo or Vismodegib, a Hedgehog Pathway Inhibitor, in Patients With Metastatic Pancreatic Cancer. J Clin Oncol.

[6] Özdemir BC, Pentcheva-Hoang T, Carstens JL, Zheng X, Wu CC, Simpson TR, Laklai H, Sugimoto H, Kahlert C, Novitskiy SV, De Jesus-Acosta A, Sharma P, Heidari P (2014). Depletion of carcinoma-associated fibroblasts and fibrosis induces immunosuppression and accelerates pancreas cancer with reduced survival. Cancer Cell.

[7] Levine B, Kroemer G (2008). Autophagy in the pathogenesis of disease. Cell.

[8] Mizushima N, Komatsu M (2011). Autophagy: renovation of cells and tissues. Cell.

[9] Dikic I, Johansen T, Kirkin V (2010). Selective autophagy in cancer development and therapy. Cancer Res.

[10] Hombach-Klonisch S, Mehrpour M, Shojaei S, Harlos C, Pitz M, Hamai A, Siemianowicz K, Likus W, Wiechec E, Toyota BD, Hoshyar R, Seyfoori A, Sepehri Z (2018). Glioblastoma and chemoresistance to alkylating agents: involvement of apoptosis, autophagy, and unfolded protein response. Pharmacol Ther.

[11] Piya S, Andreeff M, Borthakur G (2017). Targeting autophagy to overcome chemoresistance in acute myleogenous leukemia. Autophagy.

[12] Wu WK, Coffelt SB, Cho CH, Wang XJ, Lee CW, Chan FK, Yu J, Sung JJ (2012). The autophagic paradox in cancer therapy. Oncogene.

[13] White E (2015). The role for autophagy in cancer. J Clin Invest.

[14] Kondo Y, Kanzawa T, Sawaya R, Kondo S (2005). The role of autophagy in cancer development and response to therapy. Nat Rev Cancer.

[15] Redmann M, Benavides GA, Berryhill TF, Wani WY, Ouyang X, Johnson MS, Ravi S, Barnes S, Darley-Usmar VM, Zhang J (2017). Inhibition of autophagy with bafilomycin and chloroquine decreases mitochondrial quality and bioenergetic function in primary neurons. Redox Biol.

[16] Pascolo S (2016). Time to use a dose of Chloroquine as an adjuvant to anti-cancer chemotherapies. Eur J Pharmacol.

[17] Yang X, Yu DD, Yan F, Jing YY, Han ZP, Sun K, Liang L, Hou J, Wei LX (2015). The role of autophagy induced by tumor microenvironment in different cells and stages of cancer. Cell Biosci.

[18] Mizushima N, Yoshimori T, Levine B (2010). Methods in mammalian autophagy research. Cell.

[19] Yang S, Wang X, Contino G, Liesa M, Sahin E, Ying H, Bause A, Li Y, Stommel JM, Dell'antonio G, Mautner J, Tonon G, Haigis M (2011). Pancreatic cancers require autophagy for tumor growth. Genes Dev.

[20] de Duve C, de Barsy T, Poole B, Trouet A, Tulkens P, Van Hoof F (1974). Commentary. Lysosomotropic agents. Biochem Pharmacol.

[21] Homewood CA, Warhurst DC, Peters W, Baggaley VC (1972). Lysosomes, pH and the anti-malarial action of chloroquine. Nature.

[22] Rabinowitz JD, White E (2010). Autophagy and metabolism. Science.

[23] Liang XH, Jackson S, Seaman M, Brown K, Kempkes B, Hibshoosh H, Levine B (1999). Induction of autophagy and inhibition of tumorigenesis by beclin 1. Nature.

[24] Mathew R, Karp CM, Beaudoin B, Vuong N, Chen G, Chen HY, Bray K, Reddy A, Bhanot G, Gelinas C, Dipaola RS, Karantza-Wadsworth V, White E (2009). Autophagy suppresses tumorigenesis through elimination of p62. Cell.

[25] Mariño G, Niso-Santano M, Baehrecke EH, Kroemer G (2014). Self-consumption: the interplay of autophagy and apoptosis. Nat Rev Mol Cell Biol.

[26] Maycotte P, Aryal S, Cummings CT, Thorburn J, Morgan MJ, Thorburn A (2012). Chloroquine sensitizes breast cancer cells to chemotherapy independent of autophagy. Autophagy.

[27] Samaras P, Tusup M, Nguyen-Kim TD, Seifert B, Bachmann H, von Moos R, Knuth A, Pascolo S (2017). Phase I study of a chloroquine-gemcitabine combination in patients with metastatic or unresectable pancreatic cancer. Cancer Chemother Pharmacol.

[28] Verbaanderd C, Maes H, Schaaf MB, Sukhatme VP, Pantziarka P, Sukhatme V, Agostinis P, Bouche G (2017). Repurposing Drugs in Oncology (ReDO)-chloroquine and hydroxychloroquine as anti-cancer agents. Ecancermedicalscience.

[29] Cicchini M, Karantza V, Xia B (2015). Molecular pathways: autophagy in cancer--a matter of timing and context. Clin Cancer Res.

[30] Vessoni AT, Quinet A, de Andrade-Lima LC, Martins DJ, Garcia CC, Rocha CR, Vieira DB, Menck CF (2016). Chloroquine-induced glioma cells death is associated with mitochondrial membrane potential loss, but not oxidative stress. Free Radic Biol Med.

[31] Eldredge HB, Denittis A, Duhadaway JB, Chernick M, Metz R, Prendergast GC (2013). Concurrent Whole Brain Radiotherapy and Short-Course Chloroquine in Patients with Brain Metastases: A Pilot Trial. J Radiat Oncol.

[32] Molenaar RJ, Coelen RJ, Khurshed M, Roos E, Caan MW, van Linde ME, Kouwenhoven M, Bramer JA, Bovée JV, Mathôt RA, Klümpen HJ, van Laarhoven HW, van Noorden CJ (2017). Study protocol of a phase IB/II clinical trial of metformin and chloroquine in patients with IDH1-mutated or IDH2-mutated solid tumours. BMJ Open.

[33] Rojas-Puentes LL, Gonzalez-Pinedo M, Crismatt A, Ortega-Gomez A, Gamboa-Vignolle C, Nuñez-Gomez R, Dorantes-Gallareta Y, Arce-Salinas C, Arrieta O (2013). Phase II randomized, double-blind, placebo-controlled study of whole-brain irradiation with concomitant chloroquine for brain metastases. Radiat Oncol.

[34] Rosenfeld MR, Ye X, Supko JG, Desideri S, Grossman SA, Brem S, Mikkelson T, Wang D, Chang YC, Hu J, McAfee Q, Fisher J, Troxel AB (2014). A phase I/II trial of hydroxychloroquine in conjunction with radiation therapy and concurrent and adjuvant temozolomide in patients with newly diagnosed glioblastoma multiforme. Autophagy.

[35] Briceño E, Calderon A, Sotelo J (2007). Institutional experience with chloroquine as an adjuvant to the therapy for glioblastoma multiforme. Surg Neurol.

[36] Sotelo J, Briceño E, López-González MA (2006). Adding chloroquine to conventional treatment for glioblastoma multiforme: a randomized, double-blind, placebo-controlled trial. Ann Intern Med.

[37] Provenzano PP, Cuevas C, Chang AE, Goel VK, Von Hoff DD, Hingorani SR (2012). Enzymatic targeting of the stroma ablates physical barriers to treatment of pancreatic ductal adenocarcinoma. Cancer Cell.

[38] Schreiber FS, Deramaudt TB, Brunner TB, Boretti MI, Gooch KJ, Stoffers DA, Bernhard EJ, Rustgi AK (2004). Successful growth and characterization of mouse pancreatic ductal cells: functional properties of the Ki-RAS(G12V) oncogene. Gastroenterology.

